# Evaluation of CXCL9 and CXCL10 as circulating biomarkers of human cardiac allograft rejection

**DOI:** 10.1186/1471-2261-6-29

**Published:** 2006-06-19

**Authors:** Kristjan Karason, Margareta Jernås, Daniel A Hägg, Per-Arne Svensson

**Affiliations:** 1 Department of Cardiology, The Sahlgrenska Academy, Göteborgs University, SE-413 45, Göteborg, Sweden; 2 Research Centre for Endocrinology and Metabolism, Department of Metabolism and Cardiovascular Research, The Sahlgrenska Academy, Göteborgs University, SE-413 45 Göteborg, Sweden; 3 Institute of Health and Care Sciences, The Sahlgrenska Academy, Göteborgs University, SE-413 45 Göteborg, Sweden

## Abstract

**Background:**

Cardiac allograft rejection remains a significant clinical problem in the early phase after heart transplantation and requires frequent surveillance with endomyocardial biopsy. However, this is an invasive procedure, which is unpleasant for the patient and carries a certain risk. Therefore, a sensitive non-invasive biomarker of acute rejection would be desirable.

**Methods:**

Endomyocardial tissue samples and serum were obtained in connection with clinical biopsies from twenty consecutive heart transplant patients followed for six months. A rejection episode was observed in 14 patients (11 men and 3 women) and biopsies obtained before, during and after the episode were identified. Endomyocardial RNA, from three patients, matching these three points in time were analysed with DNA microarray. Genes showing up-regulation during rejection followed by normalization after the rejection episode were evaluated further with real-time RT-PCR. Finally, ELISA was performed to investigate whether change in gene-regulation during graft rejection was reflected in altered concentrations of the encoded protein in serum.

**Results:**

Three potential cardiac allograft rejection biomarker genes, chemokine (C-X-C motif) ligand 9 (CXCL9), chemokine (C-X-C motif) ligand 10 (CXCL10) and Natriuretic peptide precursor A (NPPA), from the DNA microarray analysis were selected for further evaluation. CXCL9 was significantly upregulated during rejection (p < 0.05) and CXCL10 displayed a similar pattern without reaching statistical significance. Serum levels of CXCL9 and CXCL10 were measured by ELISA in samples from 10 patients before, during and after cardiac rejection. There were no changes in CXCL9 and CXCL10 serum concentrations during cardiac rejection. Both chemokines displayed large individual variations in the selected samples, but the serum levels between the two chemokines correlated (p < 0.001).

**Conclusion:**

We conclude, that despite a distinct up-regulation of CXCL9 mRNA in human hearts during cardiac allograft rejection, this was not reflected in the serum levels of the encoded protein. Thus, in contrast to previous suggestions, serum CXCL9 does not appear to be a promising serum biomarker for cardiac allograft rejection.

## Background

Accumulating knowledge in the field of heart transplantation has led to improved patient survival and quality of life. Cardiac allograft rejection, however, continues to cause significant morbidity in the early phase after transplantation and the detection of acute rejection remains an important feature of transplant management.

The most common and reliable technique to evaluate allograft rejection is endomyocardial biopsy [1]. However, the invasive nature of the procedure is inconvenient for the patient and carries a risk for complications. These drawbacks, along with a high cost, limit the use of endomyocardial biopsy for frequent monitoring of cardiac status after transplantation. Evidently, a sensitive less-invasive technique for this purpose would be of value.

Several non-invasive methods have been studied as a tool for detecting allograft rejection, including echocardiography, electrophysiology, cytoimmunologic monitoring, radioisotopic techniques and magnetic resonance imaging (reviewed by Kemke et al [2]). Also various biochemical markers, such as neopterines [3], prolactin [4], urinary polyamines [5], beta 2-microglobulins [6] and brain natriuretic peptide (BNP) [7] have been suggested for this purpose. However, none of the above methods or markers has proven sensitive or specific enough to replace endomyocardial biopsy [8].

DNA microarray gene expression analysis and other novel technologies allowing high throughput analysis of thousands of genes are today frequently used in experimental biological studies. These techniques are more and more often being used in the study of human disease using human biopsy materials. For instance, DNA microarray have been used in the search for genes with altered gene expression in patients with prostate cancer [9], steatohepatitis [10] and leukaemia [11]. DNA microarray analysis of human heart biopsies have previously been used to investigate various heart diseases, e.g. tetralogy of Fallot [12] and atrial fibrillation [13]. The utilization of such techniques in the study of human cardiac allogaraft rejection may lead to the development of new biomarkers or novel therapies of value in monitoring and treatment.

The aim of the present study was to utilize DNA microarray analysis to search for potential biomarkers of cardiac allograft rejection.

## Methods

### Patients and biopsies

Twenty consecutive patients undergoing orthotopic heart transplantation between June 2002 and November 2003 were recruited and followed for 6 months. Immunosuppressive treatment included cytolytic induction therapy followed by a triple-drug maintenance regimen with cyclosporine or tacrolimus, azathioprine or mycophenalate mofetil and corticosteroids.

Routine endomyocardial biopsies were performed according to the following schedule: weekly during the first six weeks, every other week from week 6 to month 3 and monthly from month 3 to month 6. Biopsies were examined by experienced pathologists and graded according the 1990 working formulation of the International Society of Heart and Lung Transplantation(ISHLT) [1]. Acute cellular rejection grade 3A or higher was treated with Methylprednisolon 1 g IV daily for three days and followed up with a control biopsy 2 weeks later.

In connection with all clinical biopsies performed during the study period, two additional myocardial specimens were obtained and frozen in liquid nitrogen. At the same time, blood samples were drawn and centrifuged. Tissue samples and serum were stored at -70°C until analysis.

Episodes of rejection grade 2 or higher were identified, as well as biopsies showing grade 0 closest in time before and after each rejection. Myocardial tissue and serum samples matching these three points in time were used for analysis. In this way we defined a histopathological sequence starting with a biopsy with normal histology (before sample), followed by a rejection episode (during sample) and finally a biopsy with normal histology after the rejection episode (after sample).

The present study was approved by the regional ethics committee at Göteborg University and performed according to the declaration of Helsinki. All study subjects provided written informed consent.

### RNA isolation

RNA isolation was performed from the endomyocardial biopsies using the Chomczynski method [14] with a minor modification: during the chloroform/phenol extraction phase-lock gel tubes were used following the manufactures instructions (Eppendorf INC., Hamburg, Germany) followed by RNeasy clean-up protocol (Qiagen, Hilden, Germany). The quality of the RNA was verified by agarose gel electrophoresis before reverse transcribed into cDNA.

### DNA microarray analysis

Preparation of cRNA and hybridization to DNA microarrays was performed according to the Affymetrix Gene Chip Expression Analysis manual. Briefly, RNA was reversely transcribed into cDNA, biotin-labeled target cRNA was prepared by *in vitro *transcription (Enzo Diagnostics Inc, Farmingdale, NY) followed by hybridization to DNA microarrays, Human Genome U133A arrays, (Affymetrix, Santa Clara, CA, USA). The HU133A arrays are composed of 22.283 probe sets i.e. 12 to 16 pairs of 25-mer oligonucleotides per probe set, representing approximately 14.500 known human expressed genes. The DNA microarrays were scanned with Hewlett Packard confocal laser scanner (Hewlett Packard, GeneArray scanner G2500A).

### Data analysis

The mean target signal on each microarray was globally scaled to an average intensity of 100. Scanned output files were analyzed using Affymetrix Microarray Suite Version 5.0 software and Data Mining Tool 2.0 (Affymetrix, Santa Clara, CA, USA). An average signal for the three time points of the histopathological sequence (before, during and after rejection) was calculated from the DNA microarrays from the three different subjects analysed. The average signal values were subjected to cluster analysis using the self-organizing map (SOM) algorithm in the data mining tool software (DMT; Affymetrix). The inclusion filters for the clustering were a minimal average signal of 200 in at least one of the time points, a minimal fold change of 1.6 between at least two of the time points and a minimal maximum-minimum difference of 200 between at least two of the time points. A total of 102 genes were clustered into nine different clusters. More detailed description of the clustering parameters used in this experiment is presented as an additional file (see Additional file: 1). Genes were classified as detectable using the detection call algorithm. Any gene not displaying a present call (detectable) in all three subjects in at least one of the time points were omitted from the analysis. The identities, GeneOntology (GO) classification and function of the cluster members were investigated using the Netaffx database [15]. Tissue distribution of gene expression was analyses using the SymAtlas database [16].

### Selection of reference gene for real-time RT-PCR

The DNA microarray data was used to identify suitable reference gene for the real-time RT-PCR analysis. All probe sets for four commonly used reference genes peptidylprolyl isomerase A (cyclophilin A; PPIA), glyceraldehyde-3-phosphate dehydrogenase (GAPDH), actin beta (ACTB) and ribosomal protein, large, P0 (RPLP0), were identified using the Netaffx database. An average CV and signal ratios were calculated for the probe sets.

### Real time RT-PCR analysis

Reagents (TaqMan^® ^Reverse Transcriptase reagents and TaqMan^® ^Universal PCR Master mix) were purchased from Applied Biosystems (Foster City, CA). cDNA was synthesized with reverse transcriptase (RT) from RNA samples using random hexamers as primers. Assay-on-demand probes and primers for CXCL9 (Hs00171065_m1), CXCL10 (Hs00171042_m1), and NPPA (Hs00383236_g1) were purchased from Applied Biosystems. Conditions were used according to the manufacturer's protocol. Real time RT-PCR analysis was performed essentially as previously described [17]. In brief, amplification and detection of specific products was performed with the ABI Prism 7900 sequence detection system (Applied Biosystems) using default cycle parameters. A standard curve was plotted for each primer-probe set with a serial dilution of pooled heart cDNA in the range of 0.156 ng to 40 ng original RNA per reaction. Assay-on-demand probes and primers for human PPIA (Cyclophillin A; Hs99999904_m1) was obtained from Applied Biosystems and used as reference to normalize the expression levels between the samples. Genexpression of CXCL9, CXCL10 and NPPA were analysed in samples obtained from eight patients before, during and after cardiac rejection. All standards and samples were analyzed in triplicate.

### Serum analysis

Serum concentrations of CXCL9 and CXCL10 were determined using Quantakine ELISA kits (R&D Systems Europe Ltd., Abingdon, UK) according to the manufacturer's instructions. CRP (Latex) was determined using Tina-quant C-reactive protein high sensitive assay (HS no 1972944001; Roche Diagnostics) according to the manufacturer's instructions. Serum concentrations of CXCL9 and CXCL10 were analysed in samples obtained from 12 patients before, during and after cardiac rejection. All standards and samples were analyzed in duplicates.

### Statistical analysis

Statistical analysis was performed by ANOVA and Spearman's correlations using the AnalyzeIt 1.71 software (AnlyzeIt software Ltd, Leeds, England).

## Results

### Subjects with acute cardiac allograft rejection

A rejection episode, ISHLT grade 2 or higher, which was considered suitable for analysis, was identified in 14 patients (Table 1). This group consisted of 11 males and 3 females, with a mean age of 54 years. The patients had been referred to transplantation with the following diagnoses: dilated cardiomyopathy (n = 9), ischemic cardiomyopathy (n = 4) and hypertrophic cardiomyopathy (n = 1). The median time from transplantation to rejection was 55 days (range 20 to 104 days).

**Table 1:** Characteristics of the patients in the study. The characteristics of the patients and the different analysis performed on samples from the patients in this study. The mean age in years and the mean time from transplantation to rejection in days are shown. DNA microarray and real time RT-PCR analysis was performed on RNA from endomyocardial biopsies and ELISA analysis was performed on serum samples. * DCM = dilated cardiomyopathy, ICM = ischemic cardiomyopathy, HCM = hypertrophic cardiomyopathy.

**Patient**	Diagnosis*	**Sex**	**Age (y)**	**Rejection Time (d)**	**ISHLT**	**Analysis**		
						**Array**	**PCR**	**ELISA**
1	DCM	M	58	104	3A	X		
4	DCM	F	48	91	3		X	
5	DCM	F	38	37	2			X
6	ICM	M	66	74	3A		X	X
7	DCM	M	56	60	3A		X	X
8	DCM	M	60	77	3B	X		X
9	DCM	M	58	57	2			X
10	ICM	M	59	14	3A		X	X
11	ICM	M	63	55	3A			X
12	HCM	M	59	21	3A	X		X
13	DCM	F	37	17	3B		X	X
15	ICM	M	67	23	3A		X	X
16	DCM	M	33	55	3A		X	X
20	DCM	M	53	20	3A		X	X

Mean			54	55				

### Overall analysis of DNA microarray data

**Figure 1:**
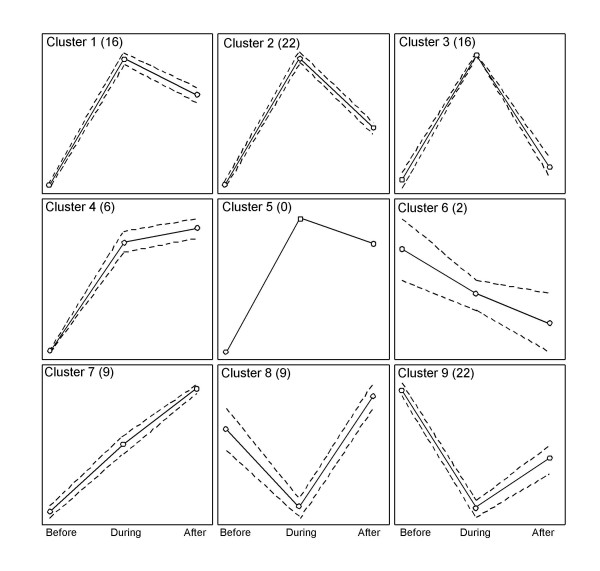
**Cluster analysis of DNA microarray data from samples before, during and after cardiac rejection**. Cluster analysis of DNA microarray data from endomyocardial biopsie samples from three subjects before, during and after cardiac rejection. Nine clusters were generated by SOM clustering containing 0–22 genes. The mean profile of the cluster members are indicated by solid lines and the dashed lines indicate standard deviation. The number of cluster members is indicated within parenthesis. Circles indicate the analysed time-points in the histopathological sequence (before, during and after rejection).

The DNA microarray analysis of endomyocardial tissue showed that approximately 44.5% (range 47% to 41%) of the genes on the DNA microarray were detectable, and a total 102 genes were defined as regulated. The raw data of the DNA microarray analysis can be found at the Gene expression omnibus (GEO) database (GSE4315) [18]. A self-organizing map (SOM) cluster analysis was used to classify regulated genes into 9 different clusters. Clusters number 5 contained no genes, whereas the remaining clusters contained between 2 and 22 genes (Figure. 1).

### Analysis of gene clusters

The genes of cluster three displayed a pattern likely to contain possible rejection markers (Figure 1). Cluster 3 contained 16 genes that were activated during rejection and returned to baseline levels after rejection (Table 2). Three genes from this cluster, chemokine (C-X-C motif) ligand 9 (CXCL9), chemokine (C-X-C motif) ligand 10 (CXCL10) and natriuretic peptide precursor A (NPPA) were selected for further evaluation based on their magnitude of activation (19-fold, 5-fold and 2.6-fold, respectively) and the fact that they encode secreted proteins/peptides (Table 2). All of the genes in cluster 3 were classified as detectable by the detection call algorithm. Also clusters 1, 2, 8 and 9 displayed an expression pattern indicating that they may contain possible rejection markers (Figure 1). However, these clusters contained few genes encoding secreted products and no genes from these clusters were selected for further evaluation (see Additional file 2).

**Table 2:** Genes included in cluster 3. Genes are presented by their Affymetrix identification code (Affy ID), gene name, gene symbol and GO cellular component classification. The mean signals from the DNA microarray analysis from the three patients during the time-points in thehistopathological sequence (before, during and after rejection) are presented. Two probe sets detecting the STAT1 and HLA-C genes are included in the cluster.

**Affy ID**	**Gene name**	**Gene symbol**	**GO cell component**	**Mean microarray signal**		
				**Before**	**During**	**After**
200887_s_at	Signal transducer and activator of transcription 1, 91 kDa	STAT1	Nucleus, cytoplasm	723	1185	719
201508_at	Insulin-like growth factor binding protein 4	IGFBP4	Extracellular	352	664	407
201762_s_at	Proteasome (prosome, macropain) activator subunit 2 (PA28 beta)	PSME2	Proteasome activator complex	370	598	424
202269_x_at	Guanylate binding protein 1, interferon-inducible, 67 kDa	GBP1	----	224	438	237
203915_at	Chemokine (C-X-C motif) ligand 9	CXCL9	Extracellular	57	1106	250
204070_at	Retinoic acid receptor responder (tazarotene induced) 3	RARRES3	----	122	425	175
204533_at	Chemokine (C-X-C motif) ligand 10	CXCL10	Extracellular	103	589	184
204806_x_at	Major histocompatibility complex, class I, F	HLA-F	----	530	1040	634
208451_s_at	Complement component 4A	C4A	Extracellular	260	589	295
209957_s_at	Natriuretic peptide precursor A	NPPA	Extracellular	1041	2780	1024
211799_x_at	Major histocompatibility complex, class I, C	HLA-C	Integral to membrane	301	670	372
214459_x_at	Major histocompatibility complex, class I, C	HLA-C	Integral to membrane	2157	3655	2553
216187_x_at	Homo sapiens Alu repeat (LNX1) mRNA sequence	----	----	472	832	551
217436_x_at	Major histocompatibility complex, class I, J	HLA-J	Integral to membrane	231	517	299
217767_at	Complement component 3	C3	Extracellular	338	531	310
HUM-ISGF3A_3_at	Signal transducer and activator of transcription 1, 91 kDa	STAT1	Nucleus, cytoplasm	390	665	357

### Identification of reference gene for real time RT-PCR analysis

Most mRNA quantification methods rely on the use of reference genes to normalize assay variability. We used a global scaling approach for the normalization of DNA microarray data (all probes on the array was used for the array-to-array normalization). Therefore, it is possible to use Affymetrix DNA microarray data to identify genes that are stably expressed and can be used as reference genes for other mRNA quantification methods [19]. Probe sets for four commonly used reference genes (GAPD, ACTB, RPLP0 and PPIA) were identified using the Netaffx database. PPIA was selected as reference gene for the real-time RT-PCR analysis based on its low average CV value (>10 %) and low between group variability (>10 %).

### Real-time RT-PCR analysis

**Figure 2:**
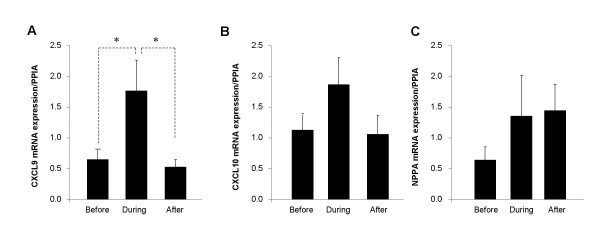
**Real-time RT-PCR analysis of gene expression before, during and after cardiac rejection**. The gene expression levels of CXCL9 (A), CXCL10 (B) and NPPA (C) in endomyocardial biopsies was determined in eight subjects using real-time RT-PCR. The different time-points in the histopathological sequence (before, during and after rejection) are indicated. Gene expression levels were relative to the reference gene peptidylprolyl isomerase A (PPIA). The results are presented as mean ± SEM. *: p < 0.05 (ANOVA).

Three genes from cluster 3, CXCL9, CXCL10 and NPPA, were selected for verification by real time PCR. The analysis was performed on samples from eight patients before, during and after rejection. The expression of CXCL9 was significantly up-regulated during rejection compared to both before and after rejection (Figure 2A; p < 0.05 and p < 0.05, respectively). The expression pattern of CXCL10 was similar to CXCL9, but the change was not significant (Figure 2B). NPPA gene expression was elevated both during and after rejection compared to the before rejection samples, however the change was not significant (Figure 2C).

### Serum analysis of CXCL9 and CXCL10 concentrations

**Figure 3:**
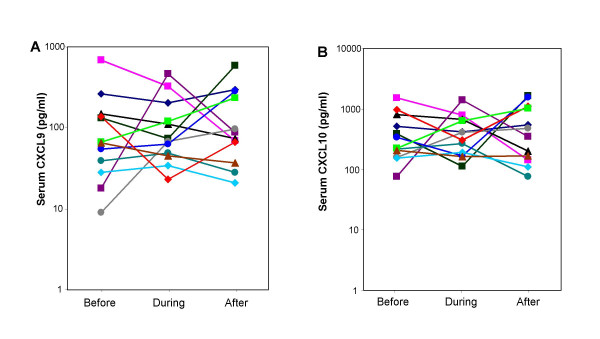
**Analysis of chemokine serum levels before, during and after cardiac rejection**. Concentrations of CXCL9 (A) and CXCL10 (B) were determined by ELISA in serum samples from twelve subjects (each represented as one line in the graph). The different time-points in the histopathological sequence (before, during and after rejection) are indicated. The results are presented as mean chemokine concentrations.

**Figure 4:**
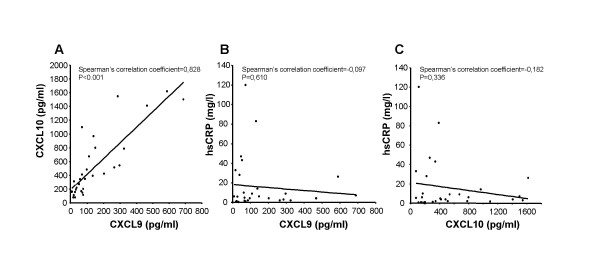
**Correlation between the serum concentrations of CXCL9, CXCL10 and hsCRP**. Concentrations of CXCL9, CXCL10 and hsCRP were determined by ELISA in serum samples from twelve subjects at different time-points in the histopathological sequence (before, during and after rejection) Correlations between CXCL9 and CXCL10 (A), CXCL9 and hsCRP (B) and CXCL10 and hsCRP (C).

Several studies have demonstrated that chemokines participate in the inflammatory response within the human transplanted heart [20,21]. Based on this and the results from the real-time RT-PCR analysis, the serum levels of CXCL9 and CXCL10 were measured by ELISA in serum samples from 10 patients before, during and after rejection. There were no changes in CXCL9 and CXCL10 serum concentrations during rejection (Figure 3A and 3B). Both these chemokines displayed large individual variations in the selected samples. However, the serum levels of these two factors correlated (Figure 4A; p < 0.001) but they did not correlate with serum high sensitive C-reactive protein (hsCRP, Figure 4B and 4C), (a general marker systemic inflammatory status; data not shown).

## Discussion

Allograft rejection remains a significant clinical problem in the first year after heart transplantation. Endomyocardial biopsy has been the most reliable method to detect rejection, but is invasive, causes discomfort to the patient and is resource demanding. Furthermore, artefacts and inter-observer variability may cause difficulties in histopathological interpretation. Several attempts have been made to identify a reliable non-invasive test for cardiac rejection, but, so far, the search for such a marker has been unsuccessful.

Despite intensive research, the mechanisms involved in organ allograft rejection, are largely unclear. DNA microarray experiments to identify mechanisms involved in cardiac rejection have previously been performed in animal models. Stegall *et al *have used DNA microarray analysis to study the Brown Norway to Lewis heterotopic heart transplant model [22]. Although many of the up-regulated genes in their study were associated with the inflammatory process, no data on the regulation of CXCL9 and CXCL10 are presented. They report an up regulation of Allograft inflammatory factor-1 (AIF1) during all of the investigated time point (3, 5 and 7 days) after heart transplantation. In our study, gene expression of AIF1 was not detectable in any of the time points investigated. Saiura *et al *has used DNA microarray analysis to investigate a mouse model of cardiac rejection. They showed that interferon (IFN)-gamma plays a central role in acutely rejected grafts by inducing several chemokines [23] and that IFN-gamma deficient mice do not have an increased expression of CXCL9 and CXCL10 during the rejection process. They also show that gene expression of macrophage inflammatory protein 1 alpha (Mip1 alpha) is increased during the rejection process both in IFN-gamma deficient and in wile type mice. However, in our study the gene expression of the human Mip1 alpha homolog (chemokine C-C motif ligand 3; CCL3) was not detectable in any of the time points investigated. The above animal models may not be representative for human heart transplants, but differences in gene expression patterns compared to our study may also be due to differences in the DNA microarrays used and/or the time points analysed.

The expression and role of CXCL9 and CXCL10 during cardiac rejection has previously been investigated both in experimental models [23-26] and in humans [20,21]. Kapoor et al has shown, in a mouse model, that the expression of CXCL9 and CXCL10 is dramatically increased during cardiac rejection and dependent upon CD8+ T cells of the recipient [24]. Similar findings have been reported by Carvalho-Gaspar *et al *[27]. Zhao *et al *showed that the expression both of CXCL9 and CXCL10 are elevated in human hearts during rejection and that the two chemokines have a differential cellular distribution in the rejecting heart. It was also proposed that the chemokine pathway might serve as a useful monitor for the treatment and prevention of cardiac rejection.

The activation of myocardial CXCL9 during the rejection process in human heart transplants, observed in the present study, is in line with previous reports. CXCL10 displayed a similar pattern, which did not reach statistical significance; possibly due to our small sample size. To further evaluate the potentiality of these chemokines as useful non-invasive biomarkers, we measured corresponding serum levels before, during and after rejection. Despite the cardiac activation of CXCL9 and CXCL10 gene expression during rejection, this was not reflected in serum levels of the encoded proteins, On the contrary, a large inter- and intra-individual variation was observed, suggesting that mechanisms other than the rejection process are of importance with respect levels of these chemokines in serum. Thus, our findings do not support the usefulness of CXL9 and CXCL10 as non-invasive markers of cardiac rejection

Cardiac natriuretic peptides, atrial natriuretic factor (ANF) and brain natriuretic peptide (BNP) have also been proposed as a potential biomarkers of cardiac allograft rejection. Previous studies have shown that serum BNP tends to increase during allograft rejection and decline following treatment [7, 28, 29], but these patterns have not proven sufficiently predictive to be clinically useful. Although the present microarray analysis indicated a moderate up-regulation of NPPA during rejection, this could not be confirmed with real time PCR. These negative findings could be explained by a small sample size and low statistical power. Furthermore, previous studies have observed a non-specific activation of natriuretic peptides up to 6 months post-transplant [30], which might obscure the relationship with early rejections.

Several markers are known to represent the systemic inflammatory status, including CRP. The CRP serum levels also displayed high variability, but did not correlate to serum CXCL9 or CXCL10 levels. This indicates that systemic inflammatory status of the patient does not directly contribute to CXCL9 and CXCL10 serum levels. A successful cardiac rejection biomarker should not only be specific to the rejection process, but also, have a limited tissue distribution profile. Bioinformatical analysis of the mRNA tissue distribution (SymAtlas database) shows that, whereas NPPA is specifically expressed in heart tissue, CXCL9 and CXCL10 are more generally expressed and may therefore be less suitable as serum markers of rejection.

The lack of success in the identification of cardiac rejection biomarkers in the current study indicate that expression profiling of immunological active cells of the heart recipient may be a better way to identify cardiac rejection biomarkers.

## Conclusion

We conclude that CXCL9 mRNA is upregulated in human hearts during rejection. However, this was not reflected in serum levels of the encoded protein. Contrary to previous suggestions [18], this indicates that CXCL9 and CXCL10 are not promising serum biomarker for this condition.

## Competing interests

The author(s) declare that they have no competing interests.

## Authors' contributions

All the authors have contributed to the design of the study, the data analysis and the writing of the manuscript. KK performed the endomyocardial biopsy procedure and patient selection. PAS performed bioinformatical and ELISA analysis. MJ carried out the DNA microarray and ELISA analysis. DAH performed the real-time RT-PCR analysis. All the authors participated in the study coordination and data interpretation. The final version of the manuscript has been read and approved by all the authors.

## Pre-publication history

The pre-publication history for this paper can be accessed here:



## Supplementary Material

Additional file 1 Parameters and settings for the SOM clustering. Click here for file

Additional file 2 Genes in clusters 1, 2, 8 and 9. Click here for file

## References

[B1] Billingham ME, Cary NR, Hammond ME, Kemnitz J, Marboe C, McCallister HA, Snovar DC, Winters GL, Zerbe A (1990). A working formulation for the standardization of nomenclature in the diagnosis of heart and lung rejection: Heart Rejection Study Group. The International Society for Heart Transplantation. J Heart Transplant.

[B2] Kemkes BM, Schutz A, Engelhardt M, Brandl U, Breuer M (1992). Noninvasive methods of rejection diagnosis after heart transplantation. J Heart Lung Transplant.

[B3] Smillie AE, Rigby RJ, Petrie JJ (1989). Monitoring the response to anti-rejection therapy with serum neopterin. Transplant Proc.

[B4] Carrier M, Russell DH, Wild JC, Emery RW, Copeland JG (1987). Prolactin as a marker of rejection in human heart transplantation. J Heart Transplant.

[B5] Carrier M, Russell DH, Davis TP, Emery RW, Copeland JG (1988). Urinary polyamines as markers of cardiac allograft rejection. A clinical evaluation. J Thorac Cardiovasc Surg.

[B6] Goldman MH, Landwehr DM, Lippman R, Hess M, Wolfgang T, Szentpetery S, Hastillo A, Mendez-Picon G, Lee HM, Lower RR (1982). Beta 2 microglobulins in rejection and cytomegalovirus infection in a cardiac transplant recipient. Transplant Proc.

[B7] Hammerer-Lercher A, Mair J, Antretter H, Ruttmann E, Poelzl G, Laufer G, Puschendorf B, Hangler H (2005). B-type natriuretic peptide as a marker of allograft rejection after heart transplantation. J Heart Lung Transplant.

[B8] Hosenpud JD (1992). Noninvasive diagnosis of cardiac allograft rejection. Another of many searches for the grail. Circulation.

[B9] Febbo PG, Richie JP, George DJ, Loda M, Manola J, Shankar S, Barnes AS, Tempany C, Catalona W, Kantoff PW, Oh WK (2005). Neoadjuvant docetaxel before radical prostatectomy in patients with high-risk localized prostate cancer. Clin Cancer Res.

[B10] Younossi ZM, Gorreta F, Ong JP, Schlauch K, Giacco LD, Elariny H, Van Meter A, Younoszai A, Goodman Z, Baranova A, Christensen A, Grant G, Chandhoke V (2005). Hepatic gene expression in patients with obesity-related non-alcoholic steatohepatitis. Liver Int.

[B11] Staber PB, Linkesch W, Zauner D, Beham-Schmid C, Guelly C, Schauer S, Sill H, Hoefler G (2004). Common alterations in gene expression and increased proliferation in recurrent acute myeloid leukemia. Oncogene.

[B12] Sharma HS, Peters TH, Moorhouse MJ, van der Spek PJ, Bogers AJ (2006). DNA microarray analysis for human congenital heart disease. Cell Biochem Biophys.

[B13] Ohki R, Yamamoto K, Ueno S, Mano H, Misawa Y, Fuse K, Ikeda U, Shimada K (2005). Gene expression profiling of human atrial myocardium with atrial fibrillation by DNA microarray analysis. Int J Cardiol.

[B14] Chomczynski P, Sacchi N (1987). Single-step method of RNA isolation by acid guanidinium thiocyanate-phenol-chloroform extraction. Anal Biochem.

[B15] http://www.affymetrix.com/index.affix.

[B16] (1987). Anal Biochem.

[B17] Svensson PA, Englund MC, Snackestrand MS, Hagg DA, Ohlsson BG, Stemme V, Mattsson-Hulten L, Thelle DS, Fagerberg B, Wiklund O, Carlsson LM, Carlsson B (2005). Regulation and splicing of scavenger receptor class B type I in human macrophages and atherosclerotic plaques. BMC Cardiovasc Disord.

[B18] http://www.ncbi.nlm.nih.gov/geo.

[B19] Gabrielsson BG, Olofsson LE, Sjogren A, Jernas M, Elander A, Lonn M, Rudemo M, Carlsson LM (2005). Evaluation of reference genes for studies of gene expression in human adipose tissue. Obes Res.

[B20] Melter M, Exeni A, Reinders ME, Fang JC, McMahon G, Ganz P, Hancock WW, Briscoe DM (2001). Expression of the chemokine receptor CXCR3 and its ligand IP-10 during human cardiac allograft rejection. Circulation.

[B21] Zhao DX, Hu Y, Miller GG, Luster AD, Mitchell RN, Libby P (2002). Differential expression of the IFN-gamma-inducible CXCR3-binding chemokines, IFN-inducible protein 10, monokine induced by IFN, and IFN-inducible T cell alpha chemoattractant in human cardiac allografts: association with cardiac allograft vasculopathy and acute rejection. J Immunol.

[B22] Stegall M, Park W, Kim D, Kremers W (2002). Gene expression during acute allograft rejection: novel statistical analysis of microarray data. Am J Transplant.

[B23] Saiura A, Kohro T, Yamamoto T, Izumi A, Wada Y, Aburatani H, Sugawara Y, Hamakubo T, Taniguchi T, Naito M, Kodama T, Makuuchi M (2002). Detection of an up-regulation of a group of chemokine genes in murine cardiac allograft in the absence of interferon-gamma by means of DNA microarray. Transplantation.

[B24] Kapoor A, Morita K, Engeman TM, Koga S, Vapnek EM, Hobart MG, Fairchild RL (2000). Early expression of interferon-gamma inducible protein 10 and monokine induced by interferon-gamma in cardiac allografts is mediated by CD8+ T cells. Transplantation.

[B25] Miura M, Morita K, Kobayashi H, Hamilton TA, Burdick MD, Strieter RM, Fairchild RL (2001). Monokine induced by IFN-gamma is a dominant factor directing T cells into murine cardiac allografts during acute rejection. J Immunol.

[B26] Yun JJ, Fischbein MP, Whiting D, Irie Y, Fishbein MC, Burdick MD, Belperio J, Strieter RM, Laks H, Berliner JA, Ardehali A (2002). The role of MIG/CXCL9 in cardiac allograft vasculopathy. Am J Pathol.

[B27] Carvalho-Gaspar M, Billing JS, Spriewald BM, Wood KJ (2005). Chemokine gene expression during allograft rejection: comparison of two quantitative PCR techniques. J Immunol Methods.

[B28] Masters RG, Davies RA, Veinot JP, Hendry PJ, Smith SJ, de Bold AJ (1999). Discoordinate modulation of natriuretic peptides during acute cardiac allograft rejection in humans. Circulation.

[B29] Ogawa T, Veinot JP, Davies RA, Haddad H, Smith SJ, Masters RG, Hendry PJ, Starling R, de Bold MK, Ponce A, Ma KK, Williams K, de Bold AJ (2005). Neuroendocrine profiling of humans receiving cardiac allografts. J Heart Lung Transplant.

[B30] O'Neill JO, McRae AT, Troughton RW, Ng K, Taylor DO, Yamani MH, Young JB, Starling RC (2005). Brain natriuretic peptide levels do not correlate with acute cellular rejection in De Novo orthotopic heart transplant recipients. J Heart Lung Transplant.

