# Supplementary material for: Evaluation of CXCL9 and CXCL10 as circulating biomarkers of human cardiac allograft rejection
Source: BMC Cardiovasc Disord. 2006 Jun 19;6:29. doi: 10.1186/1471-2261-6-29 (PMC1569871; doi:10.1186/1471-2261-6-29)
Supplement: Additional file 1 — Parameters and settings for the SOM clustering. [file 1471-2261-6-29-S1.pdf]

Additional table 1. Parameters and settings for the SOM clustering

ROW: 3
Colums: 3
Epochs: 50
Seeds: 1

Initial Neighborhood size: 5.00
Final Neighborhood size: 0.200
Initial learning rate: 0.1
Final learning rate: 0.005

Initsiation: random vectors
Neighborhood function: bubble

FILTERS:
Threshold: min 200, Max 20000
Row variation: Fold 1.6, difference 200
Row normalization: mean 0, variance 1
