# Supplementary material for: Evaluation of CXCL9 and CXCL10 as circulating biomarkers of human cardiac allograft rejection
Source: BMC Cardiovasc Disord. 2006 Jun 19;6:29. doi: 10.1186/1471-2261-6-29 (PMC1569871; doi:10.1186/1471-2261-6-29)
Supplement: Additional file 2 — Genes in clusters 1, 2, 8 and 9. [file 1471-2261-6-29-S2.pdf]

Additional table 2. Genes in clusters 1, 2, 8 and 9

Cluster 1

Affy Nr	Gene name	Gene symbol	Signal Before	During	After
201069_at	matrix metalloproteinase 2 (gelatinase A, 72kDa gelatinase, 72kDa type IV collagenase)	MMP2	268	477	445
201506_at	transforming growth factor, beta-induced, 68kDa	TGFBI	282	559	504
201666_at	tissue inhibitor of metalloproteinase 1 (erythroid potentiating activity, collagenase inhibitor)	TIMP1	482	1252	939
201721_s_at	Lysosomal-associated multispanning membrane protein-5	LAPTM5	246	615	465
201842_s_at	EGF-containing fibulin-like extracellular matrix protein 1	EFEMP1	343	552	480
202718_at	insulin-like growth factor binding protein 2, 36kDa	IGFBP2	316	660	571
203083_at	thrombospondin 2	THBS2	192	408	379
204620_s_at	chondroitin sulfate proteoglycan 2 (versican)	CSPG2	283	537	501
211071_s_at	ALL1-fused gene from chromosome 1q	AF1Q	525	868	786
211509_s_at	reticulon 4	RTN4	427	757	627
213275_x_at	cathepsin B	CTSB	340	553	493
213491_x_at	ribophorin II	RPN2	548	890	753
214629_x_at	reticulon 4	RTN4	361	608	558
215823_x_at	ring finger protein 12	RNF12	290	512	454
221731_x_at	chondroitin sulfate proteoglycan 2 (versican)	CSPG2	393	768	654
38487_at	stabilin 1	STAB1	241	501	414

Cluster 2

Affy Nr	Gene name	Gene symbol	Signal Before	During	After
200634_at	profilin 1	PFN	417	765	548
200801_x_at	actin, beta	ACTB	3302	5697	4568
201137_s_at	major histocompatibility complex, class II, DP beta 1	HLA-DPB1	342	921	612
201422_at	interferon, gamma-inducible protein 30	IFI30	188	448	294
202766_s_at	fibrillin 1 (Marfan syndrome)	FBN1	389	1004	581
204670_x_at	major histocompatibility complex, class II, DR beta 3	HLA-DRB3	860	1789	1201
208306_x_at	major histocompatibility complex, class II, DR beta	HLA-DRB3	892	2205	1561
208636_at	actinin, alpha 1	ACTN1	354	567	465
208729_x_at	major histocompatibility complex, class I, B	HLA-B	1028	1731	1354
208894_at	major histocompatibility complex, class II, DR alpha	HLA-DRA	318	948	550
209312_x_at	major histocompatibility complex, class II, DR beta 3	HLA-DRB3	761	1892	1128
209619_at	CD74 antigen (invariant polypeptide of major histocompatibility complex, class II antigen-associated)	CD74	794	2173	1366
210982_s_at	major histocompatibility complex, class II, DR alpha	HLA-DRA	396	1092	698

211529_x_at	HLA-G histocompatibility antigen, class I, G	HLA-G	562	1161	812
211990_at	major histocompatibility complex, class II, DP alpha 1	HLA-DPA1	478	1344	944
211991_s_at	major histocompatibility complex, class II, DP alpha 1	HLA-DPA1	210	476	333
212320_at	beta 5-tubulin	OK/SW-cl.56	371	635	517
213867_x_at	----		3320	5635	4227
215193_x_at	major histocompatibility complex, class II, DR beta 3	HLA-DRB3	534	1354	854
215836_s_at	protocadherin gamma subfamily C, 3	PCDHGC3	257	489	368
221875_x_at	major histocompatibility complex, class I, F	HLA-F	965	1660	1229

Cluster 8

**Affy Nr**	**Gene name**	**Gene symbol**	**Signal**		
			**Before**	**During**	**After**
204018_x_at	hemoglobin, alpha 1	HBA1	5478	3822	6151
208737_at	ATPase, H+ transporting, lysosomal 13kDa, V1 subunit G isoform 1	ATP6V1G1	418	348	571
209116_x_at	hemoglobin, beta	HBB	4842	2692	4940
209458_x_at	hemoglobin, alpha 1	HBA1	4699	3413	5951
211696_x_at	hemoglobin, beta	HBB	4843	3047	5414
211699_x_at	hemoglobin, alpha 1	HBA1	3620	2098	3753
217232_x_at	----		4521	2750	5258
217414_x_at	----		3591	1891	3905
217731_s_at	integral membrane protein 2B	ITM2B	365	337	541

Cluster 9

**Affy Nr**	**Gene name**	**Gene symbol**	**Signal**		
			**Before**	**During**	**After**
201008_s_at	thioredoxin interacting protein	TXNIP	1788	944	1315
201848_s_at	BCL2/adenovirus E1B 19kDa interacting protein 3	BNIP3	540	338	420
201849_at	BCL2/adenovirus E1B 19kDa interacting protein 3	BNIP3	824	513	695
202003_s_at	acetyl-Coenzyme A acyltransferase 2 (mitochondrial 3-oxoacyl-Coenzyme A thiolase)	ACAA2	614	299	458
202917_s_at	S100 calcium binding protein A8 (calgranulin A)	S100A8	920	147	107
203329_at	protein tyrosine phosphatase, receptor type, M	PTPRM	576	333	451
203980_at	fatty acid binding protein 4, adipocyte	FABP4	1397	718	989
204784_s_at	myeloid leukemia factor 1	MLF1	777	479	529
205882_x_at	adducin 3 (gamma)	ADD3	693	393	511
207275_s_at	fatty-acid-Coenzyme A ligase, long-chain 2	FACL2	1104	665	886
207302_at	sarcoglycan, gamma (35kDa dystrophin-associated glycoprotein)	SGCG	963	577	837
208908_s_at	calpastatin	CAST	772	449	668
209513_s_at	hypothetical protein MGC10940	MGC10940	665	392	489
210069_at	carnitine palmitoyltransferase 1B (muscle)	CPT1B	583	360	392
211569_s_at	L-3-hydroxyacyl-Coenzyme A dehydrogenase, short chain	HADHSC	663	407	531

211663_x_at	prostaglandin D2 synthase 21kDa (brain)	PTGDS	1949	1097	1635
214468_at	myosin, heavy polypeptide 6, cardiac muscle, alpha	MYH6	2513	1089	2130
214620_x_at	peptidylglycine alpha-amidating monooxygenase	PAM	1086	587	645
217329_x_at	----	----	886	484	590
218510_x_at	hypothetical protein FLJ20152	FLJ20152	1118	573	759
218869_at	pregnancy-induced growth inhibitor	OKL38	635	371	467
221932_s_at	chromosome 14 open reading frame 87	C14orf87	535	292	445
